# Increased expression of phosphorylated forms of RNA-dependent protein kinase and eukaryotic initiation factor 2*α* may signal skeletal muscle atrophy in weight-losing cancer patients

**DOI:** 10.1038/sj.bjc.6604150

**Published:** 2007-12-18

**Authors:** H L Eley, R J E Skipworth, D A C Deans, K C H Fearon, M J Tisdale

**Affiliations:** 1 Nutritional Biomedicine, School of Life and Health Sciences, Aston University, Birmingham B4 7ET, UK; 2 Tissue Injury and Repair Group, Clinical and Surgical Sciences (Surgery), University of Edinburgh, 49 Little France Crescent, Edinburgh EH16 4SB, UK

**Keywords:** muscle atrophy, cancer patients, PKR, eIF2*α*, p70^S6k^

## Abstract

Previous studies suggest that the activation (autophosphorylation) of dsRNA-dependent protein kinase (PKR) can stimulate protein degradation, and depress protein synthesis in skeletal muscle through phosphorylation of the translation initiation factor 2 (eIF2) on the *α*-subunit. To understand whether these mediators are important in muscle wasting in cancer patients, levels of the phospho forms of PKR and eIF2*α* have been determined in rectus abdominus muscle of weight losing patients with oesophago-gastric cancer, in comparison with healthy controls. Levels of both phospho PKR and phospho eIF2*α* were significantly enhanced in muscle of cancer patients with weight loss irrespective of the amount and there was a linear relationship between phosphorylation of PKR and phosphorylation of eIF2*α* (correlation coefficient 0.76, *P*=0.005). This suggests that phosphorylation of PKR led to phosphorylation of eIF2*α*. Myosin levels decreased as the weight loss increased, and there was a linear relationship between myosin expression and the extent of phosphorylation of eIF2*α* (correlation coefficient 0.77, *P*=0.004). These results suggest that phosphorylation of PKR may be an important initiator of muscle wasting in cancer patients.

Patients with cancer, especially those of the gastrointestinal tract, show a progressive wasting of skeletal muscle, which reduces both their quality of life and survival time (DeWys *et al*, 1980). Skeletal muscle atrophy is characterised by a decreased protein content, fibre diameter, force production and fatigue resistance. Muscle wasting is due to a combination of depressed protein synthesis (Emery *et al*, 1984), and elevated endogenous protein breakdown, with oxidation of the resultant amino acids (O'Keefe *et al*, 1990). In cancer patients the mechanism for the depression in protein synthesis is not known, while the increased protein degradation has been attributed to an increased expression of the ubiquitin–proteasome proteolytic pathway (Khal *et al*, 2005).

Several potential mediators of the cachectic process, including proteolysis-inducing factor (PIF) and angiotensin II (Ang II), inhibit protein synthesis in skeletal muscle (Lorite *et al*, 1997; Russell *et al*, 2006a), and also stimulate degradation, through increased activity and expression of the ubiquitin–proteasome pathway (Lorite *et al*, 2001; Sanders *et al*, 2005). We have recently shown (Eley and Tisdale, 2007) a link between the ability of PIF and Ang II to inhibit protein synthesis and increase protein degradation in murine myotubes through the dsRNA-dependent protein kinase (PKR). dsRNA-dependent protein kinase is a serine/threonine-specific protein kinase, which undergoes autophosphorylation at multiple serine and threonine residues, causing activation, in the presence of double-stranded (ds)RNA, in response to viral attack (Jammi and Beal, 2001). Both PIF and Ang II were shown to induce autophosphorylation of PKR. Activated PKR can phosphorylate several protein substrates including the *α*-subunit of the heterotrimeric translation initiation factor 2 (eIF2*α*) (deHaro *et al*, 1996). Phosphorylation of eIF2*α* inhibits continued initiation of protein synthesis by the eIF2 complex, which initiates Met tRNA binding to the 40S ribosomal subunit. During this process GTP associated with eIF2 is hydrolysed to GDP, and recycling of eIF2-GDP to eIF2-GTP requires a guanine nucleotide exchange factor eIF2B (Price and Proud, 1994). Phosphorylation of eIF2 on the *α*-subunit causes it to act as an inhibitor of eIF2B and the reduction in eIF2-GTP levels reduces general translation (Rowlands *et al*, 1998) (Figure 1). Thus activation of PKR by PIF and Ang II was responsible for the depression in protein synthesis, since transfection of myotubes with a mutant PKR incapable of autophosphorylation and induction of phosphorylation of eIF2*α*, completely attenuated the depression in protein synthesis by both agents (Eley and Tisdale, 2007). Mutation of PKR also completely attenuated the induction of protein degradation and upregulation of the ubiquitin–proteasome pathway. Induction of the ubiquitin–proteasome pathway by both PIF (Wyke and Tisdale, 2005) and Ang II (Russell *et al*, 2006b) requires activation of the transcription factor nuclear factor-*κ*B (NF-*κ*B). dsRNA-dependent protein kinase has been shown to activate the upstream kinase I*κ*B kinase (IKK) leading to degradation of the inhibitor protein I*κ*B, leading to release of NF-*κ*B, which migrates to the nucleus and induces transcriptional activation of specific genes (Zamanian-Daryoush *et al*, 2000). Myotubes containing mutant PKR showed no activation of NF-*κ*B in response to either PIF or Ang II, and no induction of the ubiquitin–proteasome pathway (Eley and Tisdale, 2007), suggesting that NF-*κ*B activity is required for the induction of ubiquitin–proteasome pathway by PKR (Figure 1). Thus activation of PKR leads potentially to both a depression of protein synthesis and an increase in protein degradation in skeletal muscle. These studies *in vitro* were also reflected by changes *in vivo* in gastrocnemius muscle of mice bearing a cachexia-inducing tumour, where levels of phosphorylated PKR and eIF2*α* were found to increase with increasing weight loss by as much as 18-fold for PKR at 25% weight loss (Eley and Tisdale, 2007).

To determine whether changes similar to those induced by PIF and Ang II also occur in human cancer cachexia, the present study examines the levels of phosphorylation of PKR and eIF2*α* in skeletal muscle of weight-losing patients with upper gastrointestinal cancer, in comparison with healthy, weight-stable subjects undergoing minor elective surgery.

## PATIENTS AND METHODS

### Cancer patients and controls

Patients provided written, informed consent, and the study was approved by the Lothian Research Ethics Committee. Twenty-nine patients with newly-diagnosed oesophago-gastric adenocarcinoma who were undergoing elective resection of their primary cancer were recruited for the study. Oesophago-gastric cancer patients have a high incidence of weight loss (DeWys *et al*, 1980) and were therefore chosen as a representative group of patients who develop cancer cachexia. Muscle biopsies were also collected from 10 healthy, weight-stable volunteers who were undergoing elective hernia surgery and who served as controls.

### Muscle biopsy

A sample of rectus abdominis muscle was obtained from the edge of the patients abdominal wound within 10 min of induction of general anaesthesia. The sample was obtained without the use of diathermy and was frozen immediately in liquid nitrogen using liquid nitrogen-resistant tubes (Corning BV, Netherlands). Samples were frozen at −70°C until analysis.

### Nutritional assessment

At the preoperative assessment, preillness stable weight was self-reported by the patient. Height was measured using a wall-mounted stadiometer with the patient standing erect without shoes. Patients were weighed on spring balance scales without shoes and wearing light clothing, and body mass index was calculated. Mid arm circumference (MAC) was measured at the midpoint between the acromion and olecranon processes. Triceps skinfold thickness (TSF) was measured with Harpenden skin callipers (Holtain, Crymych, UK). Mid arm muscle circumference (MAMC) was calculated according to the formula: MAC-[π × TSF]=MAMC. Karnofsky performance score was documented by the recruiting physician.

### Molecular biology materials

Rabbit polyclonal antibody to phospho PKR (pThr 446), was purchased from Insight Biotechnology, (Wembley, Middlesex, UK) and to total PKR (C terminus) from New England Biolabs, (Herts, UK). Rabbit polyclonal antisera to total eIF2*α* was purchased from Santa Cruz Biotechnology (CA, USA) and rabbit polyclonal antisera to phospho eIF2*α* was purchased from Abcam (Cambridge, UK). Mouse monoclonal antibody to myosin heavy chain was from Novocastra (Newcastle, UK). Rabbit polyclonal antiserum to actin was from Sigma Aldridge (Dorset, UK). Peroxidase-conjugated goat anti-rabbit antibody and peroxidase-conjugated rabbit anti-mouse antibody were purchased from Dako Ltd (Cambridge, UK). PhosphoSafe™ extraction reagent was obtained from Merck Biosciences, (Nottingham, UK). Hybond A nitrocellulose membranes and enhanced chemiluminescence (ECL) development kits were from Amersham Biosciences Ltd (Bucks, UK).

### Western blot analysis

Samples (approximately 10 mg) of muscle were homogenised in 500 *μ*l of PhosphoSafe™ Extraction Reagent and centrifuged at 15 000 g for 15 min. Samples of cytosolic protein (10 *μ*g) were resolved on 10% sodium dodecylsulphate polyacrylamide gels (6% for eIF2*α*) and transferred to 0.45 *μ*m nitrocellulose membranes, which had been blocked with 5% marvel in Tris-buffered saline, pH 7.5, at 4°C for 1–2 h. Membranes were then washed for 15 min in 0.5% Tween-buffered saline or TBS Tween prior to adding the primary antibodies. The primary antibodies were used at a dilution of 1 : 1000 except for actin (1 : 250) and anti-myosin (1 : 100). Incubation was at 4°C overnight, except for total eIF2*α* (1–2 h at room temperature). The primary antibodies were washed off the membranes for 15 min (changing the wash every 5 min), except for actin, which was washed for 45 min (changing the wash every 15 min). TBS Tween (0.1%) was used for washing phospho antibodies and total antibodies. The secondary antibodies were used at a dilution of 1 : 1000, and were washed off after 45 min. Development was by ECL and films were developed for 3–6 min. Blots were scanned by a densitometer to quantify differences.

### Statistical analysis

Western blot densitometry results are presented as means±s.e.m. for at least three replicate experiments. Differences in means between groups were determined by one-way analysis of variance, followed by Tukey–Kramer multiple comparison test. Significance level was set at *P*<0.05.

## RESULTS

The characteristics of the patients in this study is shown in Table 1. Muscle biopsies from healthy subjects undergoing elective surgery for hernia served as weight stable controls. The weight losing subjects had oesophago-gastric cancer and had a weight loss at the time of operation between 2.4 and 27.5%. As a comparison some patients with oesophago-gastric cancer without weight loss were also included.

**Figure 2:**
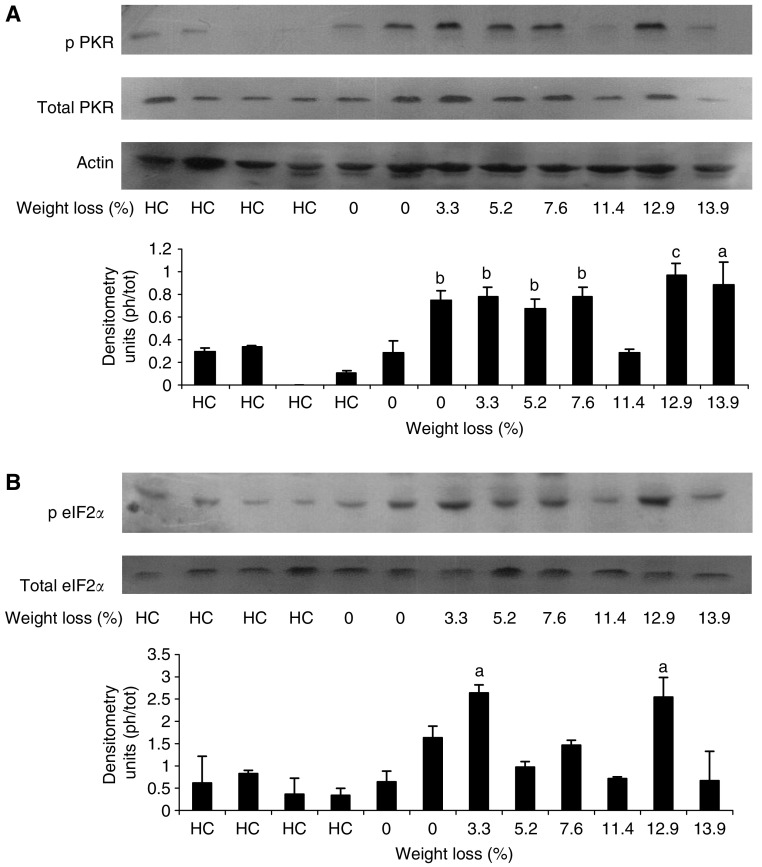
Western blots of phospho PKR (**A**) and eIF2*α* (**B**) in comparison with total PKR and eIF2*α* in rectus abdominus muscle of healthy controls (HC) and cancer patients as a function of weight loss. Actin was used as a loading control. Each lane represents muscle from an individual patient. The specificity of the antibodies is given in Patients and Methods section. A densitometric analysis of the ratio of phospho to total forms is given underneath and represents the average of three separate blots. Differences from healthy controls are shown as a, *P*<0.05 or b, *P*<0.01. c, *P*<0.001.

**Figure 3:**
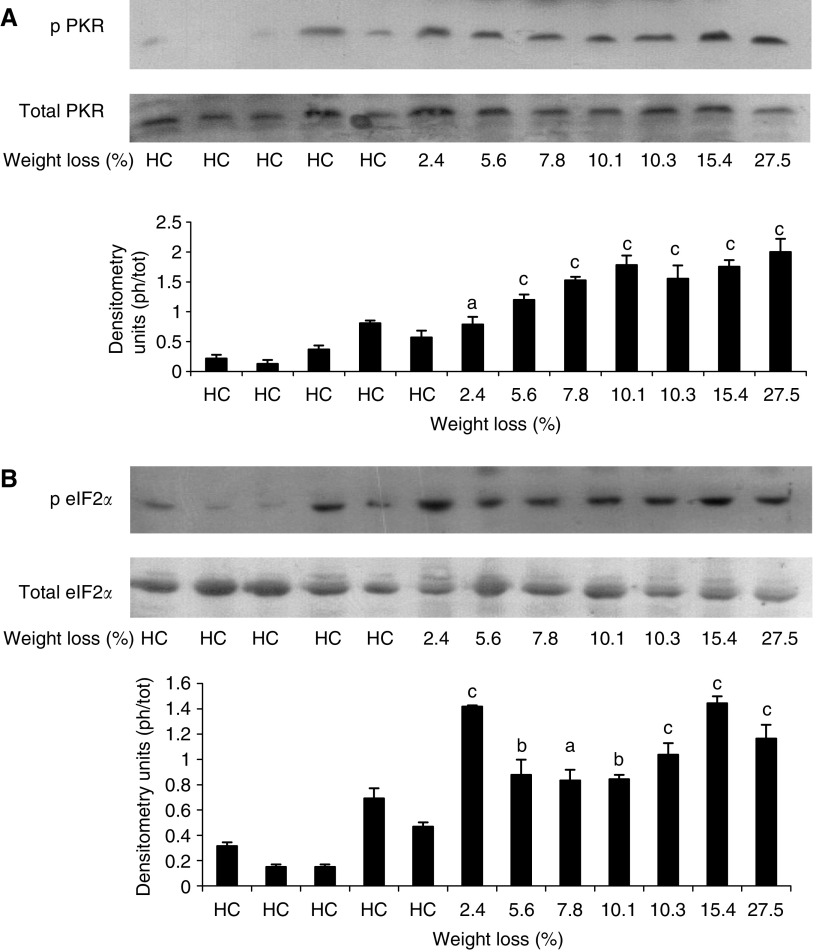
Western blots of phospho PKR (**A**) and eIF2*α* (**B**) in comparison with total PKR and eIF2*α* in rectus abdominus muscle of healthy controls (HC) and cancer patients as a function of weight loss. Actin was used as a loading control. Each lane represents muscle from an individual patient. A densitometric analysis of the ratio of phospho to total forms is given underneath and represents the average of three separate blots. Differences from healthy controls are shown as a, *P*<0.05, b, *P*<0.01 or c, *P*<0.001.

**Figure 4:**
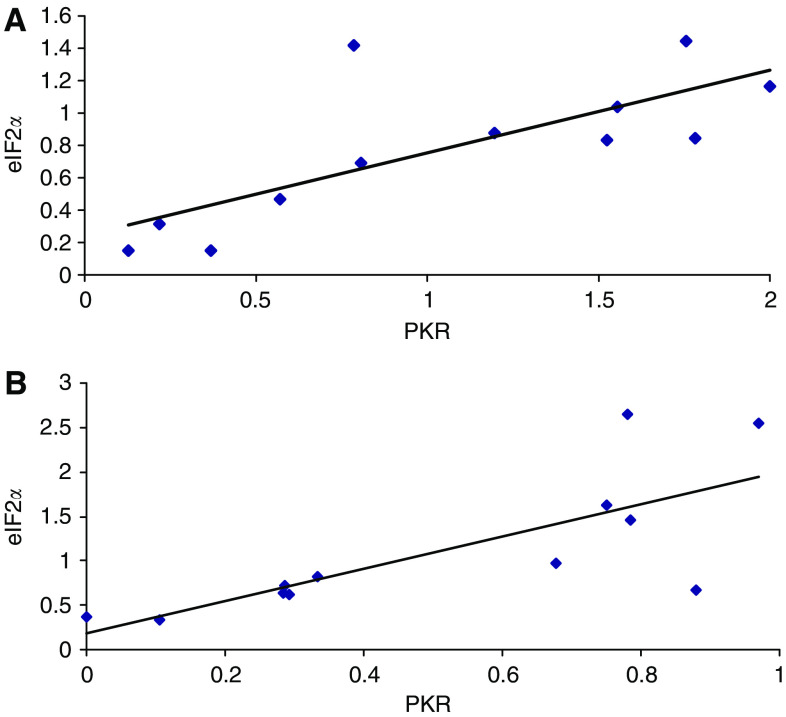
Relationship between the ratios of autophosphorylation of PKR and phospho eIF2*α* to total PKR and eIF2*α* in skeletal muscle samples from patients shown in either Figure 2A (**A**) or Figure 3B (**B**). The correlation coefficient in each case is 0.76, *P*=0.005.

**Figure 5:**
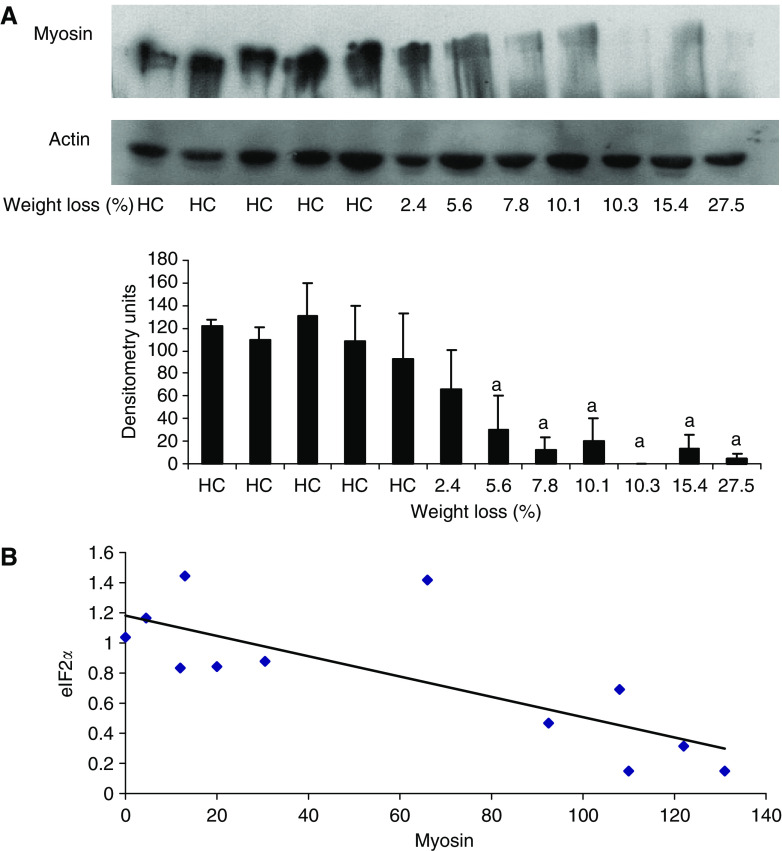
(**A**) Western blot of myosin expression in comparison with actin as a loading control in rectus abdominis muscle of the patients shown in Figure 3 as a function of weight loss. Each lane represents muscle from an individual patient. The densitometric analysis represents the average of three separate blots. Differences from healthy controls are shown as a, *P*<0.05. (**B**) Ratio of phosphorylated to total PKR *vs* the myosin levels of the samples shown in A. The correlation coefficient is 0.77, *P*<0.0004.

Western blots for the phospho and dephospho forms of PKR and eIF2*α* in rectus abdominus muscle as a function of weight loss is shown in Figures 2 and 3, which display values for different patients. While there was no major change in total PKR or eIF2*α* with weight loss, there was a significant increase in the phosphorylated forms in all patients with weight loss, which, however, did not show a tendency for increased expression with increasing weight loss. Cancer patients with no weight loss, or weight gain (Figure 2) showed the same low expression of phosphorylated PKR and eIF2*α*, as nonweight-losing normal subjects. There was a linear correlation between expression of phosphorylated PKR and phosphorylated eIF2*α* (correlation coefficient 0.76, *P*=0.005), consistent with increased PKR activity being responsible for the increased phosphorylation of eIF2 on the *α*-subunit (Figure 4). Myosin levels decreased as the weight loss increased (Figure 5A), and there was an inverse relationship between the expression of myosin in rectus abdominus muscle and the extent of phosphorylation of eIF2*α* (correlation coefficient 0.77, *P*=0.004) (Figure 5B). As previously reported (Acharyya *et al*, 2004) in skeletal muscle of mice bearing a cachexia-inducing tumour (colon 26) myosin levels decreased, while actin levels remained constant. This has been attributed to specific targeting of myosin by the ubiquitin–proteasome pathway.

## DISCUSSION

This is the first report to show an increased expression of phosphorylated PKR and eIF2*α* in the skeletal muscle of weight-losing cancer patients compared with healthy weight-stable controls. As found in the gastrocnemius muscle of weight-losing mice bearing the MAC16 tumour (Eley and Tisdale, 2007), expression of both phospho PKR and eIF2*α* increased in patients with weight loss, although there was no trend to increased expression with increasing weight loss. This suggests that the same signalling mechanism is operative in the skeletal muscle of cachectic cancer patients as that in mice with experimental cancer cachexia. Similar findings have been observed in murine myotubes in the presence of PIF or Ang II, and are thought to be responsible for the depression in protein synthesis and increase in degradation (Eley and Tisdale, 2007). dsRNA-dependent protein kinase is normally activated in response to viral attack, and the depression of protein synthesis resulting from phosphorylation of eIF2*α* constitutes one of the major ways in which viral replication is impaired (Clemens, 1997). However, PKR can also exert effects in uninfected cells and can be a potent growth inhibitory protein when activated (Chong *et al*, 1992). dsRNA-dependent protein kinase is also linked to the induction of pro-apoptotic genes by dsRNA, and may trigger cell death in response to viral infection and possible tumorigenesis (Balachandran *et al*, 1998). Activation of PKR by PIF may be responsible for its ability to induce apoptosis in murine myotubes (Smith and Tisdale, 2003). Increased apoptosis has also been observed in the skeletal muscle of rats bearing the cachexia-inducing Yoshida AH-130 ascites hepatoma (van Royen *et al*, 2000) and in the early stage of weight loss in rabbits bearing the VX2 carcinoma (Yoshida *et al*, 2001). In addition, muscle biopsies from weight losing patients with upper gastro-intestinal cancer showed a threefold increase in DNA fragmentation compared with control subjects, together with an increased PARP cleavage and decrease in MyoD protein content (Busquets *et al*, 2007). Thus activation of PKR might be responsible for the increased apoptosis in the skeletal muscle of weight-losing cancer patients contributing to muscle atrophy.

The increased phosphorylation of eIF2*α* is likely to contribute to the depression of protein synthesis in the skeletal muscle of cancer patients, through the inhibition of eIF2B and subsequent translational repression (Rowlands *et al*, 1998), as was previously observed in murine myotubes treated with PIF and Ang II (Eley and Tisdale, 2007). Phosphorylation of eIF2*α* has also been shown to be responsible for the inhibition of protein synthesis in rat liver by vasopressin (Kimball and Jefferson, 1990), and rat skeletal muscle by interleukin-1 (Cooney *et al*, 1999). Phosphorylation of PKR would also be expected to lead to an increased breakdown of myofibrillar proteins in skeletal muscle by induction of the ubiquitin–proteasome pathway (Wyke and Tisdale, 2005; Russell *et al*, 2006b) through activation of NF-*κ*B (Zamanian-Daryoush *et al*, 2000) analogous to the effect of PIF and Ang II (Eley and Tisdale, 2007).

In the current study, there was a linear relationship between activation (autophosphorylation) of PKR and phosphorylation of eIF2*α*, suggesting that PKR is responsible for this effect rather than general control of gene expression, nondepressing 2, which is expected to be activated (Anthony *et al*, 2004) by the reduction in plasma levels of amino acids in cachectic subjects (Norton *et al*, 1985). Moreover, in myotubes expressing mutant PKR there was no increase in phosphorylation of eIF2*α* in response to catabolic stimuli (Eley and Tisdale, 2007) suggesting that PKR is the major eIF2*α* kinase under such conditions.

In mice bearing the MAC16 tumour, which show a similar elevation of phospho PKR in skeletal muscle with weight loss (Eley and Tisdale, 2007), treatment with a PKR inhibitor, at a concentration which reduced levels of phospho PKR down to that found in non tumour-bearing animals, effectively attenuated the depression of body weight, through an increase in lean body mass (Eley *et al*, 2007). This was achieved through attenuation in both the depression in protein synthesis and the increase in protein degradation, as observed in murine myotubes exposed to either PIF or Ang II (Eley and Tisdale, 2007). This suggests that muscle atrophy in cachectic cancer patients may also be responsive to inhibitors of PKR.

In addition to attenuation of cachexia, treatment of mice bearing the MAC16 tumour with a PKR inhibitor also inhibited tumour growth (Eley *et al*, 2007). *In vitro* studies (unpublished) also showed sensitisation of MAC16 cells to the growth inhibitory effects of gemcitabine and 5-fluoroacil. The MAC16 tumour also shows elevated autophosphorylation of PKR and phosphorylation of eIF2*α*, which has been linked to constitutive activation of NF-*κ*B and chemoresistance (unpublished). Other studies have also shown an increased autophosphorylation of PKR and phosphorylation of eIF2*α* in human breast carcinoma cell lines, compared with nontransformed epithelial cells (Kim *et al*, 2000), and in human melanoma cells compared with nontransformed melanocytes in culture (Kim *et al*, 2002). In addition analysis of colon cancer specimens showed that transformation from normal mucosa to adenomas and carcinomas coincided with an increase in PKR expression (Kim *et al*, 2002). These results suggest a positive role of PKR in cancer progression and growth control of tumour cells. This suggests that inhibitors of PKR may not only be effective in attenuating muscle wasting in cancer patients, but may also induce antitumour effects or synergise with existing chemotherapy.

The changes that are seen may be part of a common signalling mechanism found in conditions where muscle atrophy occurs. Thus PIF has been found in the urine of weight-losing cancer patients, and when purified and administered to mice causes muscle atrophy (Cariuk *et al*, 1997). Ang II has also been linked with muscle wasting in congestive heart failure (Onder *et al*, 2002), and tumour necrosis factor-*α*, which may be linked to muscle wasting in sepsis, AIDS and parasitic infections, as well as cancer, has also been shown to activate PKR (Jeffrey *et al*, 2002). Burn injury, which also causes muscle atrophy, is also associated with an increased phosphorylation of eIF2*α* as a result of a 274% increase in phosphorylation of PKR (Kaneki *et al*, 2004). This suggests that inhibitors of PKR autophosphorylation may have a general role in the treatment of muscle atrophy.

## Figures and Tables

**Figure 1:**
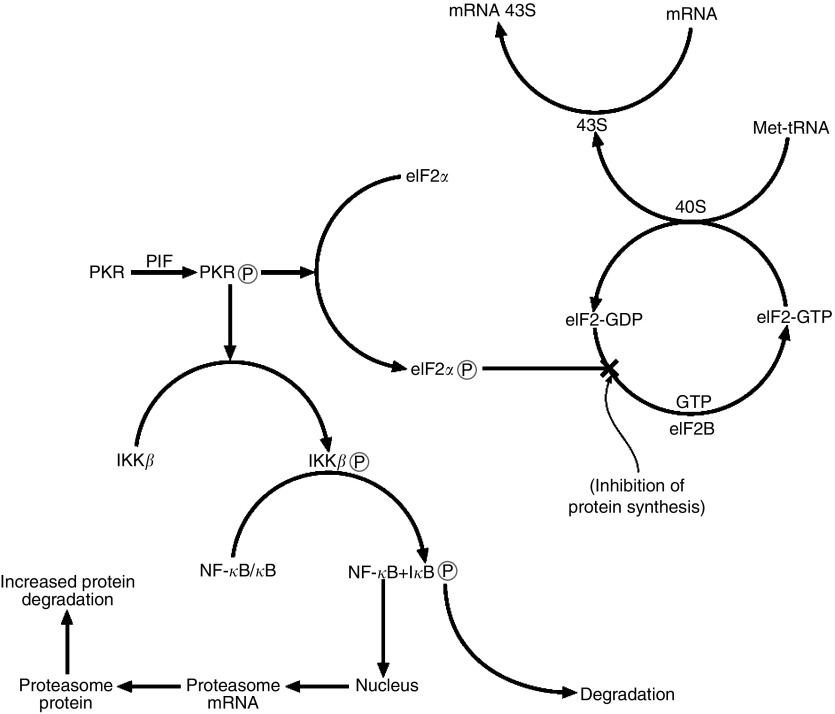
Summary of pathways leading to a depression in protein synthesis and an increase in protein degradation in skeletal muscle through activation of PKR.

**Table 1:** Demographics of the weight-losing cancer patients and weight-stable, healthy, noncancer controls

	**Healthy controls**	**Cancer patients**
Number (*n*)	9	15
		
*Sex*		
Male	9	13
Female	0	2
Age (years)	56 (41–86)	66 (49–83)
		
*Tumour site*		
Oesophageal	N/A	6
Gastric		8
		
*Histology*		
ACC	N/A	14
SCC		1
		
*Stage*		
I		3
II	N/A	3
III		6
IV		3
BMI (kg/m^2^)	28.5 (19.6–35.2)	26.1 (20.1–34.4)
MAC (cm)	32.6 (25.5–35.2)	28.9 (23.0–40.0)
TSF (mm)	15.5 (5.0–29.4)	14.4 (7.8–37.4)
MAMC (cm)	25.8 (23.8–30.5)	25.3 (18.3–30.0)
KPS	100 (100–100)	90^a^ (60–100)
Weight loss (%)	0	7.8^b^ (0.0–27.5)

Abbreviations: BMI=body mass index; KPS=Karnofsky performance score; MAC=mid-arm circumference; MAMC=mid-arm muscle circumference; TSF=triceps skinfold thickness.

Data are presented as medians with ranges in parentheses. Differences are shown from healthy controls as ^a^*P*<0.05 and ^b^*P*<0.001.
